# Coupled TLC and MALDI-TOF/MS Analyses of the Lipid Extract of the Hyperthermophilic Archaeon *Pyrococcus furiosus*

**DOI:** 10.1155/2012/957852

**Published:** 2012-11-08

**Authors:** Simona Lobasso, Patrizia Lopalco, Roberto Angelini, Rita Vitale, Harald Huber, Volker Müller, Angela Corcelli

**Affiliations:** ^1^Department of Basic Medical Sciences, University of Bari Aldo Moro, Piazza G. Cesare, 70124 Bari, Italy; ^2^Institute for Microelectronics and Microsystems, IMM-CNR, National Research Council, 73100 Lecce, Italy; ^3^Institute for Microbiology and Archaeal Centre, University of Regensburg, 93053 Regensburg, Germany; ^4^Molecular Microbiology and Bioenergetics, Institute of Molecular Biosciences, Goethe University of Frankfurt, 60438 Frankfurt/Main, Germany; ^5^Institute for Chemical-Physical Processes, IPCF-CNR, National Research Council, 70126 Bari, Italy

## Abstract

The lipidome of the marine hyperthermophilic archaeon *Pyrococcus furiosus* was studied by means of combined thin-layer chromatography and MALDI-TOF/MS analyses of the total lipid extract. 80–90% of the major polar lipids were represented by archaeol lipids (diethers) and the remaining part by caldarchaeol lipids (tetraethers). The direct analysis of lipids on chromatography plate showed the presence of the diphytanylglycerol analogues of phosphatidylinositol and phosphatidylglycerol, the *N*-acetylglucosamine-diphytanylglycerol phosphate plus some caldarchaeol lipids different from those previously described. In addition, evidence for the presence of the dimeric ether lipid cardiolipin is reported, suggesting that cardiolipins are ubiquitous in archaea.

## 1. Introduction

*Pyrococcus furiosus* is an aquatic anaerobic hyperthermophilic archaeon, originally isolated from geothermally heated marine sediments near Vulcano Island, Italy [1]. It can grow between 70°C and 103°C, with an optimum temperature of 100°C, and between pH 5 and pH 9 (with an optimum at pH 7). The cells appear as regular cocci of 0.8 *μ*m to 1.5 *μ*m diameters with monopolar polytrichous flagellation and cellular envelope composed of a glycoprotein distinguishing them from bacteria. 

*P. furiosus* is unique among its kind in that it can use a wide range of compounds as carbon source, such as peptides and carbohydrates [2]. Unlike other hyperthermophiles, it does not need elemental sulphur for growth [3]. It is also notable that some of its enzymes are tungsten dependent, a very rare element which is to be found in biological systems [4]. 

*P. furiosus* has an unusual and intriguingly simple respiratory system, which obtains energy by reducing protons to hydrogen gas and uses this energy to create a proton gradient across its cell membrane, thereby driving ATP synthesis. The A_1 _A_0_ ATP synthase from *P. furiosus* has been recently isolated and its three-dimensional structure was analyzed by electron microscopy [5]. The sequencing of the complete *P. furiosus *genome was completed in 2001; it is 1.91 Million *bp* long and contains approximately 2228 predicted genes [6]. 

**Figure 1:**
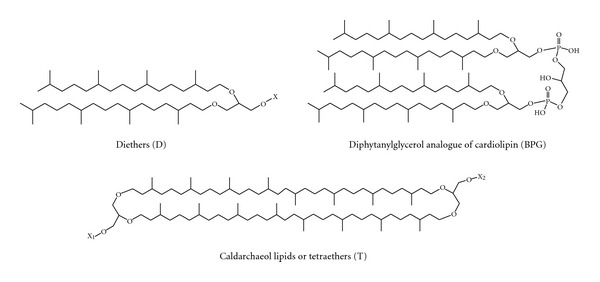
General structures of diphytanylglycerol-derived lipids diethers (D) and tetraethers (T). The diphytanylglycerol lipid core of membrane lipids of Archaea is also named archaeol. X, X_1_, and X_2_ represent the head groups of polar lipids.

One of the most peculiar features of archaea is represented by the structural properties of their membrane lipids. Archaeal membrane lipids are constituted by mostly saturated phytanyl chains in ether linkage to glycerol carbons with *sn*-2,3 configuration, forming diether (archaeol) and membrane-spanning tetraether (caldarchaeol) lipids (Figure 1) [7–9]. Furthermore, ether analogues of cardiolipins, bisphosphatidylglycerol (or BPG) and glycosyl-cardiolipins, have been found in the membranes of extremely halophilic archaea [10–13]. The archaeal bisphosphatidylglycerol is a dimeric phospholipid containing four identical branched C_20_ lipid chains, as it is synthesized at the expense of C_20_ C_20_ phosphatidylglycerol based on the 2,3-di-*O*-phytanyl-*sn*-glycerol diether lipid core (PG) [14, 15]. Recently ether lipid cardiolipin variants, constituted by different combinations of C_20_ and C_25_ isopranoid chains, have been found in two extremely haloalkaliphilic archaea *Natronococcus occultus *and *Natronococcus amylolyticus *[16]. Studies conducted so far have not revealed the presence of diphytanylglycerol analogues of cardiolipin in hyperthermophilic archaea. 

Previous studies on lipids of hyperthermophilic archaea of the Thermococcaceae family have been reported [17–20]. Although some lipid structures of *P. furiosus* were proposed on the basis of FAB-MS studies [20], the individual lipids have not been isolated nor have complete structures to be determined. 

In the present work we have reexamined the lipids of the hyperthermophilic archaeon *P. furiosus* with modern analytical methods with the aim of enriching our knowledge of the lipidome of this microorganism and in particular of checking for the possible presence of novel archaeal cardiolipins. Here we show that the dimeric phospholipid cardiolipin is present in hyperthermophilic archaea and report additional novel findings on membrane diether and tetraether lipids of this microorganism.

## 2. Material and Methods

### 2.1. Materials

9-Aminoacridine hemihydrate was purchased from Acros Organics (Morris Plains, NJ, USA). The following commercial glycerophospholipids (used as standards): 1,1′,2,2′-tetratetradecanoyl cardiolipin, 1,1′,2,2′-tetra-(9*Z*-octadecenoyl) cardiolipin, 1,2-ditetradecanoyl-*sn*-glycero-3-phosphate, and 1,2-ditetradecanoyl-*sn*-glycero-3-phospho-(1′-*rac*-glycerol), 1,2-ditetradecanoyl-*sn*-glycero-3-phospho-L-serine, 1,2-di-(9*Z*-hexadecenoyl)-*sn*-glycero-3-phosphoethanolamine, were purchased from Avanti Polar Lipids, Inc. (Alabaster, AL, USA). In addition, the archaeal cardiolipins bisphosphatidylglycerol (BPG), isolated from *Hbt. salinarum, *and the glycosylated cardiolipin (2′-sulfo)Man*pα*1-2Glc*pα*1-1-[*sn*-2,3-di-*O*-phytanylglycerol]-6-[phospho-*sn*-2,3-di-*O*-phytanylglycerol] (S-DGD-5-PA), isolated from *Halorubrum sp.* MdS1 strain [15], were used as standard in the present study. All organic solvents used in the lipid extraction and MS analyses were commercially distilled and of the highest available purity and were purchased from Sigma Aldrich, J.T. Baker or Carlo Erba. HPTLC and TLC plates (HPTLC Silica gel 60 A, aluminium plates and TLC Silica gel 60 A glass plates), obtained from Merck, were washed twice with chloroform/methanol (1 : 1, v/v) and activated at 180°C before use.

### 2.2. Microorganism and Growth Conditions

*P. furiosus *(DSM 3638) was obtained from the Deutsche Sammlung für Mikroorganismen und Zellkulturen, Braunschweig, Germany. *P. furiosus* was grown in a 300 L fermenter at 98°C in the medium described [21]. The fermenter was pressurized to 2 bar with N_2_/CO_2_ (80 : 20). The gas flow through was adjusted to 1–7 L/min, depending on the growth phase. Growth was monitored by cell counts. Cells were harvested in the late exponential growth phase by centrifugation (10,000 ×g, 20 min, 4°C). The pellets were stored at −80°C.

### 2.3. Lipid Extraction

Total lipids were extracted using the Bligh and Dyer method [22], as modified for extreme halophiles [23]; the extracts were carefully dried under N_2_ before weighing and then dissolved in chloroform (10 mg/mL). 

### 2.4. High-Performance Thin-Layer Chromatography (HPTLC)

Total lipid extracts were analyzed by HPTLC (Merck 10 × 20 cm, aluminium back) with Solvent A (chloroform/methanol/90% acetic acid, 65 : 4 : 35, v/v). Lipid detection was carried out by spraying with 5% sulfuric acid in water, followed by charring at 180°C for 7-8 min [23], or alternatively spraying the plate with a solution of primuline [24] and detecting lipid upon excitation by UV light (336 nm). Furthermore, the following stainings were performed in order to identify the lipid classes present in the TLC bands: (a) Molybdenum-Blue Sigma spray reagent for phospholipids; (b) Azure-A/sulfuric acid for sulfatides and sulfoglycolipids; (c) ninhydrin in acetone/lutidine (9 : 1) for free amino groups [23].

### 2.5. Isolation and Purification of Individual Lipids from the Total Extract

The lipid components of the total lipid extract of *P. furiosus* were separated by preparative TLC (Merck 20 × 20 cm × 0.2 mm thick layer, glass plates) in Solvent A. Lipids were visualized by staining with iodine vapour and were eluted and recovered from the scraped silica, as previously described [10]. Isolated and purified phospholipids were dissolved in chloroform at the concentration of 1 mg/mL.

### 2.6. Preparation of Lipid Samples in Solution for MALDI-TOF/MS

Samples were prepared as previously described [25]. Briefly, the total lipid extract (10 mg/mL) and individual lipid components (1-2 mg/mL) were diluted from 20 to 200 *μ*L with isopropanol/acetonitrile (60 : 40, v/v). Next, 10 *μ*L of diluted sample was mixed with 10 *μ*L of the matrix 9-aminoacridine (10 mg/mL; dissolved in isopropanol/acetonitrile (60 : 40, v/v)). Then 0.25 *μ*L of the mixture was spotted on the MALDI target (Micro Scout Plate, MSP 96 ground steel target).

The protein pellet resulting from the lipid extraction of *P. furiosus* cells was recovered and directly analyzed by MALDI-TOF/MS avoiding the second lipid extraction, as recently described [26]. 

### 2.7. Coupling of MALDI-TOF/MS to HPTLC 

The procedure was carried out according to Fuchs et al. [24], with minor modifications, cutting the HPTLC plates in pieces (about 4 × 8 cm in size) containing all the lipids present in the total extract. These pieces, which corresponded to a single lane of HPTLC, were then fixed onto the MALDI target with double-sided adhesive tape. Three small droplets of saturated matrix (9-aminoacridine) solution (in total 1.5 *μ*L) were then deposited onto each point, obtaining a continuous deposition along the HPTLC lane. Then the matrix deposition points were numbered and each of them was assigned to HPTLC band areas of interest by comparing the retention factors resulting from staining by lipid charring. Then all the matrix deposition points were directly analyzed with MALDI-TOF/MS. Although for each TLC band a certain number of mass spectra were acquired, only the most representative spectra are shown.

### 2.8. MALDI-TOF Mass Spectrometry

MALDI-TOF mass spectra were acquired on a Bruker Microflex RLF mass spectrometer (Bruker Daltonics, Bremen, Germany). The system utilizes a pulsed nitrogen laser, emitting at 337 nm, the extraction voltage was 20 kV, and gated matrix suppression was applied to prevent detector saturation. 999 single laser shots (sum of 3 × 333) were averaged for each mass spectrum. The laser fluence was kept about 10% above threshold to have a good signal-to-noise ratio. In particular for the analysis of the bands on the HPTLC plates, the laser fluence was 20% more than in the analysis of the lipids in solution; in fact a major fluence is needed to desorb lipids from the silica. All spectra were acquired in reflector mode using the delayed pulsed extraction; only spectra acquired in negative ion mode are shown in this study. Spectral mass resolutions and signal-to-noise ratios were determined by the software for the instrument, “Flex Analysis 3.3.65” (Bruker Daltonics).

Post Source Decay (PSD) spectra were acquired on a Bruker Autoflex mass spectrometer (Bruker Daltonics), as previously described [27]. Briefly, the precursor ions were isolated using a time ion selector. The fragment ions were refocused onto the detector by stepping the voltage applied to the reflectron in appropriate increments. This was done automatically by using the “FAST” (“fragment analysis and structural TOF”) subroutine of the Flex Analysis software.

A mix containing 1,1′,2,2′-tetratetradecanoyl cardiolipin, 1,1′2,2′-tetra-(9*Z*-octadecenoyl) cardiolipin, 1,2-ditetradecanoyl-*sn*-glycero-3-phosphate, 1,2-ditetradecanoyl-*sn*-glycero-3-phospho-(1′-rac-glycerol), 1,2-ditetradecanoyl-*sn*-glycero-3-phospho-L-serine, 1,2-di-(9*Z*-hexadecenoyl)-*sn*-glycero-3-phosphoethanolamine, and the archaeal glycosylated cardiolipin (2′-sulfo)Man*pα*1-2Glc*pα*1-1-[sn-2,3-di-*O*-phytanylglycerol]-6-[phospho-*sn*-2,3-di-*O*-phytanylglycerol] (S-DGD-5-PA) was always spotted next to the sample as external standard and an external calibration was performed before each measurement; the mass range of the authentic standards is 590–1770 a.m.u.

## 3. Results and Discussion

**Figure 2:**
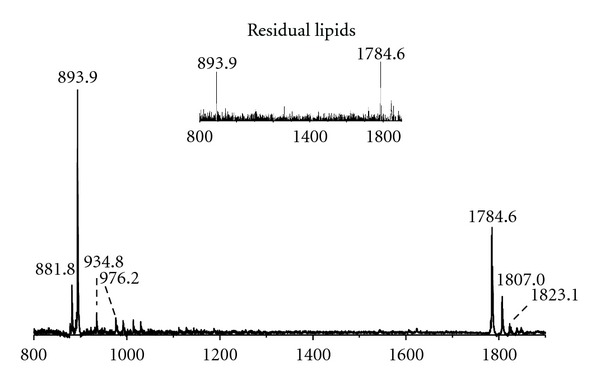
MALDI-TOF/MS lipid profiles of the total lipid extract of *Pyrococcus furiosus *(DSM3638) acquired in the negative ion mode using 9-aminoacridine as the matrix. The lipid components assigned to the main *m/z* ion peaks are unsaturated diphytanylglycerol analogue of phosphatidylinositol (*uns*PI), [M-H]^−^ at *m/z* 881.8; diphytanylglycerol analogue of phosphatidylinositol (PI), [M-H]^−^ at *m/z* 893.8; *N*-acetylglucosamine-diphytanylglycerol phosphate (*N*-acetyl-hexose-P-D) [M-H]^−^ at *m/z* 934.8; diglycosyl-diphytanylglycerol (DGD) [M-H]^−^ at *m/z* 976.2. The ion peak [M-H]^−^ at *m/z* 1784.6 is attributable to a caldarchaeol lipid, with its sodium and potassium adducts at *m/z *1807.0 and 1823.1, respectively. The detailed list of detected ion peaks is shown in Table 1. *Inset:* MALDI-TOF/MS lipid analyses in intact mode (as described in ref. [26]) of the residual pellet after the lipid extraction.

Figure 2 shows the MALDI-TOF mass spectrum of the total lipid extract of *P. furiosus* acquired in the negative ion mode, using 9-aminoacridine as matrix. Since MALDI ionization is quite soft, molecular ions of lipids are predominant in mass spectra by using the proper matrix. As MALDI-TOF/MS allows ionization and transfer of the lipid sample from the solid phase to gas phase, it is similar to FAB-MS, previously used to study the lipids of *P. furiosus* [20]. The peaks in the spectrum can be grouped in two main *m/z* ranges: signals attributable to diphytanylglycerol lipids, also named archaeol lipids, or simply diethers (D), in the range 700–1000, and those attributable to caldarchaeol lipids (i.e., tetraethers, T), typically in the range 1500–2000. The high *m/z* range is also the area of the mass spectrum where cardiolipins and complex glycosylated-cardiolipins can be found. The main peak at *m/z *893.9 corresponds to an inositol-diphytanylglycerol phosphate, the diphytanylglycerol analogue of phosphatidylinositol (PI), which is found in several Archaea and also previously observed in *Pyrococcus* species [18, 20]. The signal at *m/z* 881.8 can be assigned to the unsaturated inositol-diphytanylglycerol phosphate (*uns*PI), containing double bonds in its archaeol moiety, as detailed discussed in the following (Figure 4(a)). The peak at *m/z *935.0 is likely to represent an *N-*acetylglucosamine-diphytanylglycerol phosphate (abbreviated in the following as *N*-acetyl-hexose-P-D), while that at *m/z* 976.2 represents a glycolipid derivative of diphytanylglycerol carrying two sugar units in the polar head (diglycosyl diether, DGD), both previously described in FAB mass spectrum of *P. furiosus* [20]. The peak at *m/z* 1784.6 present in the higher *m/z* range of the MALDI-TOF mass spectrum (Figure 2) is attributable to a caldarchaeol or tetraether lipid; the peaks at *m/z* 1807.0 and 1823.1 correspond to sodium and potassium adducts, respectively. This caldarchaeol lipid has a molecular mass slightly higher than that of the diglycosyl phosphatidylglycerol tetraether (hexose_2_-PG-T, see structure in Figure 6) giving rise to the peak at *m/z* 1778.5, previously found in FAB mass spectrum of *P. furiosus* [20].

The MALDI-TOF/MS profile of residual lipids which is still associated with the heterogeneous protein pellet left after the lipid extraction of *P. furiosus* cells is also reported in the inset of Figure 2. It can be seen that the two main peaks at *m/z *1784.6 and 893.9 are still visible in the lipid profile of protein pellet after the lipid extraction, indicating that lipid extraction is not complete; although we have not estimated the quantity of residual lipids left behind after the first lipid extraction, as the intensity of the peak at *m/z* 1784.6, corresponding to the caldarchaeol, is higher than that of the peak at *m/z* 893.9, we conclude that it is more difficult to extract caldarchaeol lipids than diether lipids, as previously reported [28].

Furthermore MALDI-TOF mass spectrum of the total lipid extract of *P. furiosus *was also acquired in positive ion mode: only minor signals attributable to fragments of lipid branched-chains were detected in the *m/z* range 400–600, while no peaks were present in the *m/z* range of phospholipids and glycolipids (not shown). The lack of signals in the (+) MALDI-TOF mass spectrum suggests that the diether analogue of phosphatidylcholine is absent in *P. furiosus* and that most, if not all, lipid components are acidic. 

**Figure 3:**
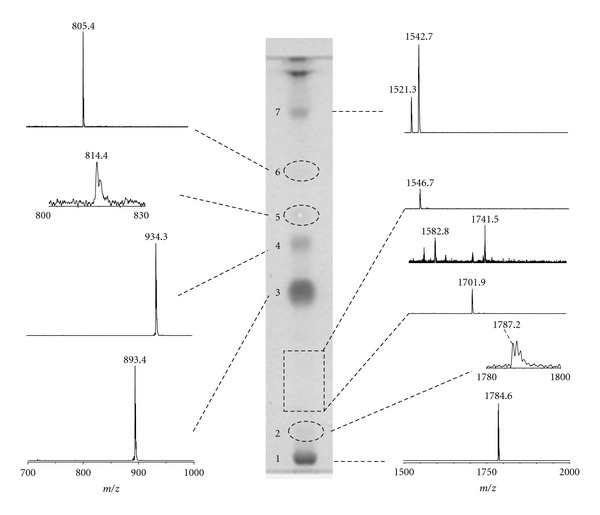
Coupled MALDI-TOF/MS and HPTLC analyses of the total lipid extract of *P. furiosus*. The total lipid extract was applied on the TLC plate (200 *μ*g) in two lanes; the lipid bands in one lane were charred after spraying with 5% sulphuric acid, while 9-aminoacridine was applied manually along the other lane obtaining a continuous deposition. The main bands on the TLC plate after charring are shown in the centre of the picture, while, on the right and the left of the TLC, are shown the negative spectra obtained by MALDI scanning of the lane covered with the matrix. Dashed lines on the TLC plate were used to mark pale lipid bands.

In order to deeper investigate on the lipid components present in the total lipid extract of *P. furiosus, *we performed a detailed staining analysis after HPTLC and isolated some of the lipid components by preparative TLC. The TLC lipid profile of *P. furiosus *is shown in Figure 3. There are four main lipid bands (1, 3, 4, and 7), plus neutral pigments visible at the solvent front in the HPTLC plate. Individual lipid components of *P. furiosus* were identified not only by their responses to specific lipid staining, but also by MALDI-TOF/MS analysis of purified lipid components by preparative TLC; in particular the fragmentation behaviour of some polar lipids of *P. furiosus* was also investigated by using Post Source Decay (PSD) mass spectrometry analysis.

All the major lipid components were found to be positive to blue molybdenum staining (not shown). The only exception was the pale band marked by an asterisk. No lipid component was found to be positive to Azure-A or ninhydrin-staining (not shown); therefore the main lipids of *P. furiosus* are phospholipids, while do not contain sulphate or amino groups in their polar moieties. 

The band 1 (in *R*_*f*_ order) is a tetraether phospholipid or caldarchaeol. Two diether phosphoglycolipids have been identified in bands 3 and 4, inositol-diphytanylglycerol phosphate, and *N*-acetylglucosamine-diphytanylglycerol phosphate, respectively, giving rise to MALDI-TOF/MS signals at *m/z* 893.9 and 935.0, respectively, both observed in the mass spectrum of the total lipid extract, previously shown in Figure 2. In addition, close to the solvent front, the pale band 7 corresponds to a phospholipid, having an *R*_*f*_ value similar to that of the authentic standard diphytanylglycerol analogue of bisphosphatidylglycerol BPG (not shown), previously described for extremely halophilic and haloalkaliphilic archaea [10–13, 16]. 

**Figure 4:**
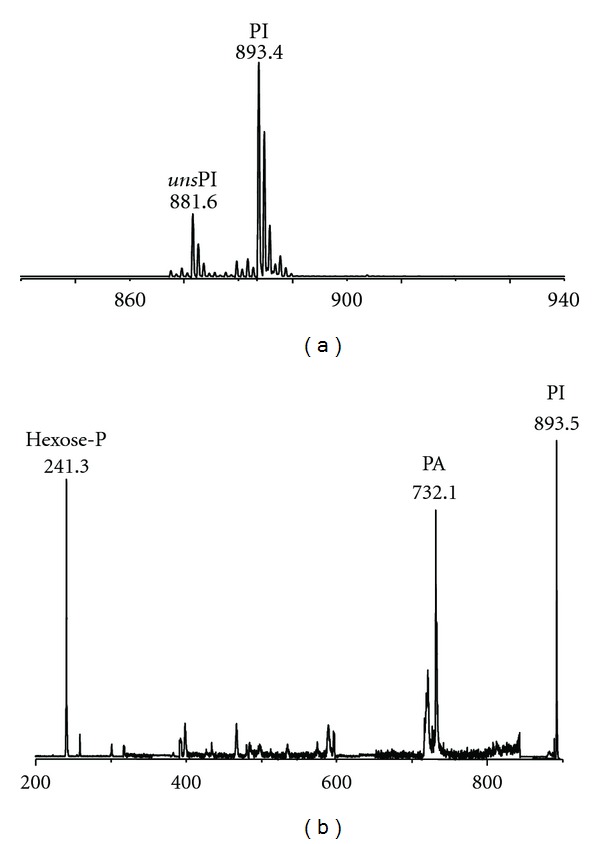
MALDI-TOF mass (a) and PSD fragment ion (b) spectra of band 3 of *P. furiosus*. (a): the lipid components present in band 3 (see TLC in Figure 3) were isolated and purified from the total lipid extract of the *P. furiosus* by preparative TLC. Peaks corresponding to the molecular ions of diphytanylglycerol analogue of phosphatidylinositol (PI) at* m/z* 893.4 and of the corresponding unsaturated species (*uns*PI) at *m/z* 881.6. (b): peaks corresponding to the molecular ion of PIat* m/z* 893.45 plus the ion fragments corresponding to the diphytanylglycerol analogues of PA (*m/z* 732.1) and to the sugar-phosphate residue (*m/z* 241.3).

**Figure 5:**
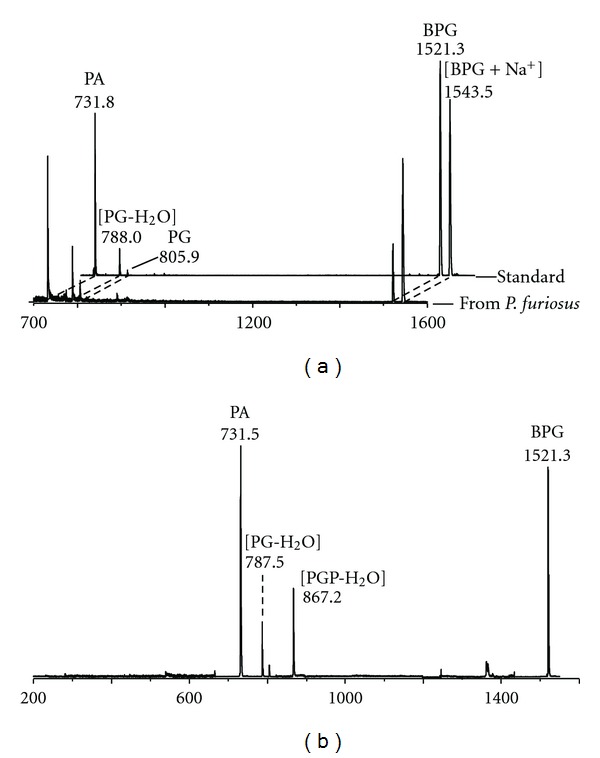
(a) Comparison of the MALDI-TOF mass spectra of the lipid component in band 7 of *P. furiosus* (see TLC in Figure 3) and the authentic standard BPG (i.e., diphytanylglycerol analogue of bisphosphatidylglycerol) isolated from *Halobacterium salinarum*. (b) PSD fragment ion spectrum of the diphytanylglycerol analogue of BPG of *P. furiosus. *Abbreviations: PA: diphytanylglycerol analogue of phosphatidic acid; PG: diphytanylglycerol analogue of phosphatidylglycerol; PGP: diphytanylglycerol analogue of phosphatidylglycerolphosphate.

**Figure 6:**
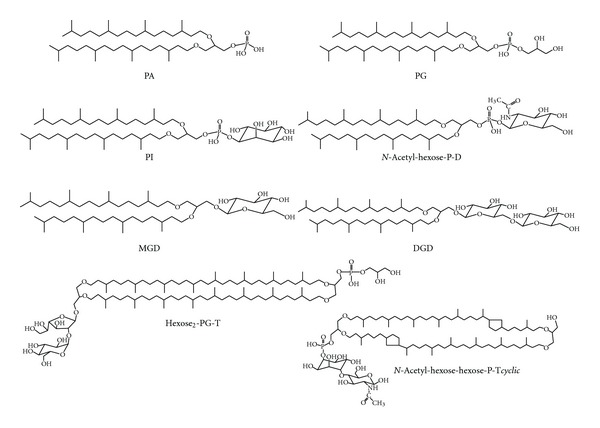
Polar membrane lipids of *P. furiosus*. Abbreviations used in the present study (see Table 1) to indicate phospholipids and glycolipids present in the membrane of *P. furiosus* always refer to diphytanylglycerol derived lipids. Two caldarchaeol lipids are shown: the main caldarchaeol (named *hexose *_2_ *-PG-T*) found in previous literature studies [20] and the structure proposed by us for the lipid component (named *N-acetyl-hexose-hexose-P-Tcyclic*) giving raise to the peak at *m/z* 1741.5 in MALDI-TOF/MS analysis.

The individual lipid components of *P. furiosus *were also analysed directly on HPTLC plate by MALDI-TOF/MS, as described in detail in experimental procedures. Selected negative ion mass spectra, obtained during the MALDI scanning of the main bands visible on the TLC, plate are shown in Figure 3. Proceeding by *R*_*f*_ order, from the bottom to the top of the plate:

(1) the analysis of the first band (band 1) revealed a main signal at *m/z* 1784.6, which has been tentatively attributed to a caldarchaeol phospholipid possibly having the general structure of (hexose-P)_2_-T. The lipid component corresponding to band 1 has been isolated by preparative TLC and its structure was further investigated by PSD fragment ion spectra analysis (not shown). Results confirmed the presence of the caldarchaeol lipid core in the molecule, but the precise nature and location of sugar residues in the structure have not been determined;
(2) in band 2, an area of the plate located immediately upon the first band, we obtained a mass spectrum with a small signal at *m/z* 1787.2 that could correspond to a lipid compound closely related to the lipid present in band 1; furthermore, going from the bottom toward the solvent front, between the bands 2 and 3, traces of other caldarchaeol lipids, giving rise to signals at *m/z *1701.9, 1741.5, and 1546.7 (in *R*_*f*_ order), have been found. These three last caldarchaeol lipids are structurally similar; the compound giving rise to the molecular ion at *m/z* 1741.5 could correspond to the acetylated form of the *m/z* 1701.9 (see proposed structure in Figure 6) lipid and it is indeed more mobile in the TLC. The lipid yielding the ion peak at *m/z* 1546.7 could correspond to a caldarchaeol bearing only one sugar unit;
(3) the MALDI-TOF/MS analysis of the major lipid band (band 3) showed a signal at *m/z* 893.4, which is assigned to the diphytanylglycerol analogue of phosphatidylinositol (PI). The mass spectrum of isolated band 3 (Figure 4(a)) showed, together with the main peak at *m/z* 893.4, a signal at *m/z* 881.6 which can be assigned to an unsaturated diphytanylglycerol analogue of phosphatidylinositol (named *uns*PI), containing six double bonds in its archaeol moiety (hexaunsaturated archaeol). Furthermore, Figure 4(b) shows the PSD fragment ion spectrum of the diphytanylglycerol analogue of phosphatidylinositol at *m/z *893.4; as an abundant fragment at *m/z* 731.2, diagnostic for the structure of the diether analogue of phosphatidic acid (PA), was found, we can exclude the presence of another lipid having identical *m/z* (i.e., glucosyl-3-phosphate diether), previously found in *Pyrococcus* strain AN1 [18].
(4) the analysis of the band 4 showed a signal at *m/z* 934.3, which can be assigned to *N*-acetylglucosamine-diphytanylglycerol phosphate, according to previous FAB-MS analysis of *P. furiosus* [20]; as in the MALDI-TOF mass spectrum recorded at the level of band 4 the fragment at *m/z* 731.2 was not present, we conclude that the phosphate is not directly linked to the archaeol lipid core. Furthermore, the MALDI-TOF mass spectrum of the lipid component in band 4 (not shown), isolated and purified from the total lipid extract by preparative TLC, revealed the presence (at *m/z* 922.6) of minor amounts of an unsaturated analogue of the *N*-acetylglucosamine-diphytanylglycerol phosphate (abbreviated as *uns-N-*acetyl-hexose-P-D), having six double bonds in its archaeol moiety.
(5) The spectrum of the pale band 5 showed a very small signal at *m/z* 814.2, which could correspond to a monoglycosyl diphytanylglycerol (monoglycosyl diether, MGD); this compound could be the precursor of the glycolipid present in band 4;
(6) the analysis of the area corresponding to band 6 on the TLC plate revealed a small amount of the diphytanylglycerol analogue of phosphatidylglycerol (PG), having the signal of the molecular ion at *m/z* 805.4;
(7) the chromatographic behaviour coupled to information given by MALDI-TOF/MS analysis allowed an easy precise identification of lipid component in band 7. The MS analysis of the band 7, having the same *R*_*f*_ of diphytanylglycerol analogue of BPG, confirmed the presence of BPG in the lipid profile of *P. furiosus*, with signals at *m/z* 1521.3 and 1542.7, corresponding to [M-H]^−^ and [M-H+Na^+^]^−^, respectively. The complete MALDI-TOF mass spectrum of the lipid component in band 7 is shown in Figure 5(a), together with the mass spectrum of the authentic BPG standard isolated from *Halobacterium salinarum*. It is concluded that the archaeal analogue of cardiolipin of *Pyrococcus* is identical to that present in *Halobacterium salinarum* and other extremely halophilic microorganisms [10–13, 16]. Furthermore, the PSD fragment ion spectrum of the lipid component in band 7 is shown in Figure 5(b): it can be seen that the ion fragments corresponding to the diphytanylglycerol analogues of PA (*m/z* 731.5), PG-H_2_O (*m/z* 787.5), PG (*m/z* 805.9), and PGP-H2O (*m/z* 867.2) are present. All these peaks are diagnostic for the structure of the diphytanylglycerol analogue of bisphosphatidylglycerol, or ether lipid cardiolipin. Noteworthy, the PSD fragmentation pattern of the diphytanylglycerol analogue of cardiolipin is similar to that of mitochondrial cardiolipin [26].

In conclusion, the present study describes the presence of three different kinds of membrane lipids in the *P. furiosus*: diether lipids, dimeric diether lipids and tetraether lipids (see Figure 1). Structures of all lipids, described through the present study are illustrated in Figure 6.

Caldarchaeol lipids of *P. furiosus* found in the present study are different from those previously reported [20]. The main caldarchaeol lipid exhibits a main MALDI-TOF/MS peak at *m/z* 1784.6, tentatively assigned to an (hexose-P)_2_-T, which do not correspond to any previous described caldarchaeol lipid of *Pyrococcus* or other hyperthermophilic archaea. We have accurately searched data in the literature and in lipid databases without finding a lipid candidate having the mass matching with that obtained in the present study. The finding of different caldarchaeols likely depends on differences in microorganism growth conditions. The precise structure of the main caldarchaeol of *P. furiosus* here found is presently under study, also with the help of analytical approaches different from mass spectrometry.

In the microorganism growth conditions of the present study diphytanylglycerol phosphoglycolipids are the most abundant lipid components of *P. furiosus*; unsaturated phosphoglycolipids have been also observed in minor proportions. The diphytanylglycerol analogue of phosphatidylglycerol is only a minor diether lipid. Altogether, diether lipids represent the majority of the lipids in the total lipid extract. This finding is in agreement with previous studies [18, 20]. Other reports have shown that the proportions between archaeol and caldarchaeol lipids can be reversed, as are profoundly influenced by the temperature and conditions of growth [29].

Only minute amounts of the diphytanylglycerol analogue of bisphosphatidylglycerol or cardiolipin have been found in the lipid extract in our experimental conditions. This is the first report describing the presence of dimeric diphytanylglycerol lipids in a hyperthermophilic archaeon. Novel cardiolipins have been recently described in uncultured methane-metabolizing archaea [30]; we also found a diphytanylglycerol analogue of bisphosphatidylglycerol in cultured *Methanocaldococcus jannaschii* (unpublished data). A number of studies report that prokaryotes contain variable amounts of cardiolipin depending on their physiologic state and experimental conditions of lipid analyses. It has been previously shown that the diether cardiolipin archaeal analogue is located in membrane domains performing bioenergetic functions, in analogy with bacteria [11, 31–34]. In consideration of the peculiarity of its respiratory system, in future studies it will be interesting to study the role of cardiolipin on the membrane enzymes involved in the bioenergetics of *P. furiosus.*

## Figures and Tables

**Table 1:** Assignments of *m/z* values detected in the negative ion mode MALDI-TOF mass spectra and PSD analyses of the *P. furiosus* total lipid extract to various lipid components. Lipids are indicated as abbreviations.

Observed [M–H]^−^ signals (*m/z*)	Assignments	Calculated [M–H]^−^ (*m/z*)
1784.6	(Hexose-P)_2_-T	1784.3
1741.5	*N*-Acetyl-hexose-hexose-P-T*cyclic *	1741.4
1701.9	Hexose_2_-P-T *cyclic *	1702.4
1543.5	BPG plus Na^+^	1542.3
1521.3	BPG	1520.3
976.2	DGD	975.8
934.8	*N*-Acetyl-hexose-P-D	934.7
922.6	*uns*-*N*-Acetyl-hexose-P-D	922.6
893.8	PI	893.7
881.6	*uns*PI	881.5
867.2	PGP minus H_2_O	868.6
814.4	MGD	813.7
805.7	PG	805.7
787.5	PG minus H_2_O	787.7
731.8	PA	731.6
241.3	Hexose-P minus H_2_O	242.0

Abbreviations—D: diether or archaeol; T: tetraether or caldarchaeol; T*cyclic*: caldarchaeol with cyclopentane rings; P: phosphate group; *uns*: unsaturated branched-chains, 3 double bonds for chain; *N*-acetyl-hexose: *N*-acetylglucosamine; PI: diphytanylglycerol analogue of phosphatidylinositol; BPG: diphytanylglycerol analogue of bisphosphatidylglycerol (or ether lipid cardiolipin); PG: diphytanylglycerol analogue of phosphatidylglycerol; PGP: diphytanylglycerol analogue of phosphatidylglycerol-phosphate; PA: diphytanylglycerol analogue of phosphatidic acid; MGD: monoglycosyl-diphytanylglycerol; DGD: diglycosyl-diphytanylglycerol.
